# Tier-specific contextualised high-intensity running profiles in the English Premier League: more on-ball movement at the top

**DOI:** 10.5114/biolsport.2023.118020

**Published:** 2022-07-21

**Authors:** Wonwoo Ju, Richard Hawkins, Dominic Doran, Antonio Gómez-Díaz, Andrés Martín-García, Mark Evans, Andy Laws, and Paul S Bradley

**Affiliations:** 1 The Research Institution for Sport and Exercise Sciences at Liverpool John Moores University, Liverpool, England, UK; 2 Football Medicine and Science Department at Manchester United Football Club, Manchester, UK; 3 Clube de Regatas do Flamengo, Rio de Janeiro, Brazil; 4 FC Barcelona Sports Performance Department, Barcelona, Spain; 5 Department of Computer Science, Liverpool John Moores University, Liverpool, UK; 6 Football Science Consultant, UK

**Keywords:** Football, Match performance, Physical-tactical data, Standard, Ranking

## Abstract

The present study aimed to determine the physical-tactical profiles of elite football teams and individual players according to final league rankings. A total of 50 English Premier League matches (n = 100 match and 583 player observations) were analysed by coding the player’s physical-tactical actions through synchronising tracking data and video. Final league rankings were categorised into Tiers: (A) 1^st^–5^th^ ranking (n = 25), (B) 6^th^–10^th^ ranking (n = 26), (C) 11^th^–15^th^ ranking (n = 26), and (D) 16^th^–20^th^ ranking (n = 23). One-way analyses of variance were used to compare match performances between different Tiers, and effect size (ES) was determined for the meaningfulness of the difference. Tier A teams covered 39–51% more high-intensity distance for ‘Move to Receive/Exploit Space’ (ES: 1.3–1.6, P < 0.01) and ‘Run with Ball’ (ES: 0.9–1.0, P < 0.05) than Tier C and D, and 23–94% more distance for ‘Over/Underlap’ (ES: 1.0, P < 0.01), ‘Run in Behind/Penetrate’ (ES: 0.7, P < 0.05), and ‘Break into Box’ (ES: 0.9, P < 0.05) compared to Tier C. Central and Wide Defensive Players in Tier A covered 65–551% more high-intensity ‘Move to Receive/Exploit Space’ distance compared to other Tiers (ES: 0.6–1.0, P < 0.01). Moreover, the additional options within the physical-tactical actions and zonal differences unveiled more meaningful insights into ‘HOW’ the top Tier teams physically and tactically perform. Thus, the amalgamated physical-tactical data help improve our understanding of a team’s playing style relative to their competitive standard.

## INTRODUCTION

The energetic demands of football (soccer) during a match can be indirectly quantified via time-motion analysis, which can provide valuable data to applied coaching staff [1]. Although a plethora of research has quantified the physical demands of elite football during matchplay [2–5], limited evidence exists on the relationship between success in football and physical performance [3, 6]. This ambiguity seems to be due to limited consideration of the tactical context pertaining to the physical data as tactical scenarios during match-play are one of the factors modulating the physical actions that occur in football [7].

Previous research that has examined associations between physical data and final league rankings have demonstrated that lower-ranked clubs ran greater total distance in high-speed running during match-play with higher-ranked teams performing more when in possession of the ball [8–11]. Although success in football appears to be more likely associated with greater high-intensity in-possession actions whilst maintaining possession to create more space and attacking threats, it is unknown what types of tactical actions are performed pertaining to high-intensity efforts [10, 11]. Therefore, to improve our understanding of team success, tactical context should be tagged alongside the physical metrics. Furthermore, such limited relationships are possibly due to the methodological approach most previous research has adopted [3, 4, 10, 12]. For instance, only individual player performances rather than collective team performances are considered. As football is a team sport where physical and tactical performances of players are affected by not only opponent but also teammate’s activities [13], more research is warranted to understand if team performance characteristics are informative when trying to gain insights into the determinants of success.

Technical performances, rather than physical performance per se (e.g., high-intensity running distance), seem to be better indications to predict a team’s success and to demarcate between various team standards and/or league rankings in elite football [4, 14]. Higher-ranked clubs tend to have a greater number of shots on target, ball touches, and passes, as well as a higher percentage of pass accuracy compared to lower-ranked clubs [14, 15]. However, using technical metrics in isolation is still one-dimensional and insufficient to understand a team’s success and to differentiate between team standards and/or league rankings in football given the fact that players’ performances are impacted by the combination of physical, tactical, technical, and psychological as well as contextual parameters [13, 16]. Some studies have attempted to integrate physical metrics with technical data, but the method they used was not an integration but an aggregation of such performances within their results [10, 12].

Currently, a systematic integrated approach that can contextualise physical metrics with key tactical purposes has been established [17]; however, this approach still does not include technical performance. This is due to this novel approach still requiring a manual coding process, which is labour intensive [18]. Hence, amalgamating high-intensity running activities with the key tactical purpose of the action could be a starting point [19]. Despite this shortcoming, the novel approach appears to be a possible solution to better understand a team’s success through discriminating between team standards since various physical-tactical patterns of teams/players according to their final league ranking may be identified. Therefore, this present study aimed to determine the physical-tactical profiles of elite football teams and individual players with reference to final league rankings to identify associations between success and physical-tactical data alongside technical metrics.

## MATERIALS AND METHODS

### Team and player data

Match physical-tactical data were collated from the 2018–19 English Premier League (EPL) season using an integrated approach and a new filter developed for this research. Players’ motions were captured by cameras placed at roof level during matches and their physical-tactical activities were coded using the integrated approach. The validity and reliability of this approach and the novel filter were previously verified [17], from which detailed methodological information can be found. Using the new filter, high-intensity activities reaching speeds > 19.8 km · h^−1^ for a minimal dwell time of 1 s were isolated [20].

The researcher underwent 350 hours of coding to analyse 50 competitive games. This consisted of the total number of 388 individual outfield players across 1,265 player observations within 20 different teams. All of the player’s physical-tactical actions for each match were summarised to analyse team performances (those who were subbed in or out were included; *n* = 100 match observations). However, regarding an individual player’s analysis, only outfield players who had completed the entire match in the same position were included (n = 583 player observations). This consisted of 179 Central Defensive players (CDP), 147 Wide Defensive players (WDP), 167 Central Midfield players (CMP), 54 Wide Offensive players (WOP), and 36 Central Offensive players (COP). All data were analysed for the duration of each half, including stoppage time. Prior to analysis, all original data were anonymised to ensure confidentiality. Research approval was given by the local Ethics Committee of the appropriate institution.

### Match control and data balance

Matches were randomly chosen while concurrently controlling several contextual factors (e.g., phases of season, location, and team/opposition standard) to enhance the scientific rigor of the research design [12]. Matches were omitted if goal differential was > 3 and a player dismissal occurred since these impact match running performances [21, 22].

### League ranking categorisation into tiers

The classification of final league rankings was determined using four Tiers: (A) 1^st^–5^th^ ranking (*n* = 25 match observations), (B) 6^th^–10^th^ ranking (*n* = 26 match observations), (C) 11^th^–15^th^ ranking (*n* = 26 match observations), (D) 16^th^–20^th^ ranking (*n* = 23 match observations). Categorising league ranking is challenging due to inter- and intra-season variations of team performance; however, a generic process was applied to explore the physical-tactical performances by different Tiers [10].

### The integrated approach used to quantify match performance

Two main coding categories were used: tactical actions and additional options to make this approach more systematic (Table 1). Isolated high-intensity actions were synchronised with wide-angle video footage of all players throughout matches to categorise the tactical purpose of each action. All coding occurred using QuickTime Player (Apple Inc, Cupertino, California) to view video footage of high-intensity efforts and then categorise their tactical actions.

**TABLE 1:** Physical-tactical variables and additional options (direction and/or different situational options). adapted from Ju et al. [17].

Variables	Description	Additional Options
**In Possession**		

Run with Ball	Player moves with the ball either dribbling with small touches or running at speed with fewer ball touches.	Drive forward/diagonal/lateral (Central) Run down/up channel (Wide) Run into channel (Central to Wide) Drive inside (Wide to Central)

Over/Underlap	Player runs from behind to in front of the player on the ball or receiving the ball.	Run down channel (Wide) Run into channel (Central to Wide)

Push up Pitch	Player moves up the pitch to play offside and/or to squeeze to a higher line.	Move forward/diagonal (Central) Run down channel (Wide) Move into channel (Central to Wide) Move inside (Wide to Central)

Break into Box	Player enters the opposition’s penalty box.	Towards the central zone in the box (Central) Towards one of the wide zones in the box (Wide) Towards the central zone through a wide zone in the box (Wide to Central) Within the box

Run in Behind/Penetrate	Player attacks space behind, overtakes and/or unbalances the opposition defence.	Drive forward/diagonal (Central) Run down channel (Wide) Run into channel (Central to Wide) Drive inside (Wide to Central)

Move to Receive/ Exploit Space	Player moves to receive a pass from a teammate and/or to create/exploit space.	Move forward/diagonal (Central) Move backward/diagonal/lateral (Central) Run down/up channel (Wide) Run into channel (Central to Wide) Drive inside (Wide to Central)

Support Play	Player supports from behind/level by trying to engage in offensive/transition play.	Drive forward/diagonal (Central) Run down channel (Wide) Run into channel (Central to Wide) Drive inside (Wide to Central)

**Out of Possession**		

Close Down/Press	Player runs directly towards opposition player on or receiving the ball, or towards space or players that are not a viable passing option.	Towards the player on the ball (after ball touch) Towards the player receiving the ball (before ball touch) Space/a player

Interception	Player cuts out the ball during the transition of a pass.	Intercept the ball in offensive third Intercept the ball in offensive-mid third Intercept the ball in defensive-mid third Intercept the ball in defensive third

Recovery Run	Player runs back toward their own goal to be goal side of the ball when out of position.	Run back towards own goal (ball behind) Run back towards own goal from attacking/set play (ball still in front) Ball passed over top/down side (opposition closer to the ball)

Covering	Player moves to cover space or an opposition player while remaining goal side of the ball.	Space/a player Long Ball/Pass (> 25 m; not beaten by opposition)

Close Down/Press	Player runs directly towards opposition player on or receiving the ball, or towards space or players that are not a viable passing option.	Towards the player on the ball (after ball touch) Towards the player receiving the ball (before ball touch) Space/a player

**Unclassifiable**		

Other	All other variables that could not be classified by the above.	Each additional option also has ‘Other’.

The coding process was as follows: high-intensity actions with one tactical action were classified as a single action (*n* = 27,054) with dual tactical actions being classified as a hybrid action (*n* = 4,718). High-intensity actions with more than three tactical actions were coded as ‘Other’ (*n* = 3,398). If the high-intensity effort consists of 70–90% the primary with 10–30% of the secondary action, it was classified as a hybrid action. However, if it is made up of 50–60% of the primary with 40–50% of the secondary action, then it was classified as ‘Other’. Since hybrid actions are an amalgamation of the primary and the secondary actions [19], all hybrid actions were analysed with the former to simplify data outputs. Additional options were also analysed using the descriptions (Table 1). The intra-rater reliability for the additional options (*n* = 241) revealed 88% of agreement with the kappa statistic value of 0.87, interpreted as a strong intra-observer reliability [23].

### Technical data

Technical tracking data from the matches analysed were collected from an established company (OPTA Sports, London, United Kingdom). The reliability of this system has been verified [24]. Technical events such as the number of shots, shots on target, ball touches, passes, crosses, dribbles, long passes, accurate long passes, and interceptions as well as pass accuracy were analysed. All individual data for each match were summed up to represent team performances.

### Statistical analyses

Data are presented as the mean ± standard deviation. All statistical analyses were conducted using IBM SPSS Statistics for Mac OS X, version 26 (IBM Corp., Armonk, N.Y., USA). Data normality was verified by Shapiro-Wilk and Kolmogorov-Smirnov tests. One-way analyses of variance were used to compare match performances by each Tier with Tukey’s post hoc test used to determine localised differences. Statistical significance was set at *P* < 0.05. Effect sizes (ES) for the meaningfulness of the difference were determined as follows: trivial (≤ 0.2), small (> 0.2–0.6), moderate (> 0.6–1.2), large (> 1.2–2.0), very large (> 2.0–4.0) and extremely large (>4.0) [25]. The Pearson correlation coefficient was used for correlation analyses. According to Hopkins et al. [26], the magnitudes of the correlation coefficients were regarded as trivial (r ≤ 0.1), small (r > 0.1–0.3), moderate (r > 0.3–0.5), large (r > 0.5–0.7), very large (r > 0.7–0.9), and nearly perfect (r > 0.9). The coefficients of variation (CV) were analysed for match-to-match variabilities of team performances [27].

## RESULTS

### Contextualised high-intensity distance according to tiers

**FIG. 1:**
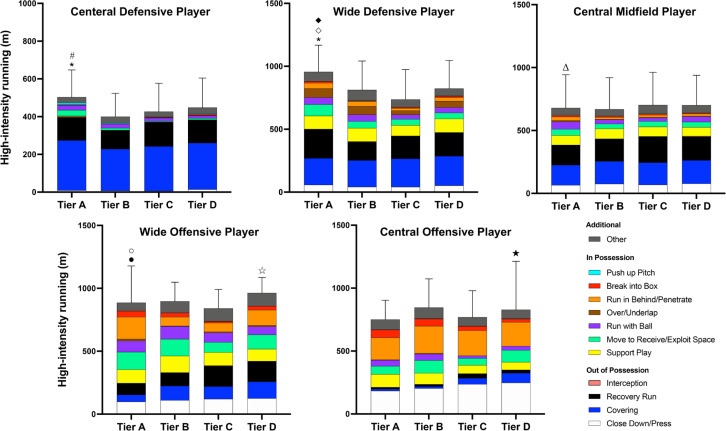
Contextualised high-intensity distances covered by various positions in different Tiers. *Greater distance covered for ‘Move to Receive/Exploit Space’ than Tier A, B, and C (P < 0.01). #Greater distance covered for ‘Push up Pitch’ than Tier C (P < 0.05). ◇Greater distance covered for ‘Over/Underlap’ than Tier C (P < 0.05). ◆Greater distance covered for ‘Recovery Run’ than Tier B (P < 0.05). ΔGreater distance covered for ‘Run with Ball’ than Tier B and C (P < 0.05). ●Greater distance covered for ‘Run in Behind/Penetrate’ than Tier B and C (P < 0.01). ○Greater distance covered for ‘Break into Box’ than Tier C (P < 0.01). ☆Greater distance covered for ‘Covering’ than Tier A (P < 0.05). ★Greater distance covered for ‘Covering’ than Tier A and B (P < 0.05). The volume of ‘Interception’ and ‘Push up Pitch’ distances was relatively small; thus, they are invisible on the figure.

Tier A teams covered 34% more high-intensity distance when in possession than Tier C and D (ES: 1.4–1.6, P < 0.01, Table 2) whilst covering 39–51% more high-intensity distance for ‘Move to Receive/Exploit Space’ (ES: 1.3–1.6, *P* < 0.01) and ‘Run with Ball’ (ES: 0.9–1.0, *P* < 0.05) compared to Tier C and D, and 23–94% more distance for ‘Over/Underlap’ (ES: 1.0, *P* < 0.01), ‘Run in Behind/Penetrate’ (ES: 0.7, *P* < 0.05), and ‘Break into Box’ (ES: 0.9, *P* < 0.05) compared to Tier C. Contextualised high-intensity distances covered across various positions in different Tiers are shown in Figure 1.

**TABLE 2:** High-intensity distances across various tiers.

Tier	A	B	C	D	Difference and Effect Size
Total	7778 ± 1039	7242 ± 962	7078 ± 1233	7153 ± 1190	# (ES: 0.1–0.6)
IP	3277 ± 533	2914 ± 742	2439 ± 647	2447 ± 512	A > C*/D* (ES: 1.4–1.6) A > B# (ES: 0.6)
OOP	3835 ± 838	3653 ± 798	3968 ± 878	4057 ± 952	# (ES: 0.1–0.5)
Other	666 ± 195	675 ± 191	671 ± 173	649 ± 239	# (ES: 0.0–0.1)

ES: Effect sizes: trivial (≤ 0.2), small (> 0.2–0.6), moderate (> 0.6–1.2), large (> 1.2–2.0), very large (> 2.0–4.0) and extremely large (>4.0) [25]. Asterisk (*) denotes differences (*P* < 0.05); Hash (#) denotes no differences (*P* > 0.05). IP: In Possession, OOP: Out of Possession. Values are represented as means and standard deviations (m).

### Additional options across different tiers

Figure 2 illustrates the comparison of additional options for in- (A, B, and C) and out-of-possession (D and E) variables between Tiers. In possession, teams in Tier A performed 88–118% more high-intensity actions in the central zone whilst moving backwards than other Tiers (ES: 1.0–1.1, *P* < 0.01). Clubs in Tier A also executed 54–78% and 25–43% more high-intensity actions for ‘Run with Ball’ and ‘Run in Behind/Penetrate’ in the central zone, respectively, than other Tiers (ES: 0.7–1.2, *P* < 0.05). The average numbers of physical-tactical actions produced by various Tiers across different zones are illustrated in Figure 3 (A for in-possession and B for out-of-possession variables).

**FIG. 2a:**
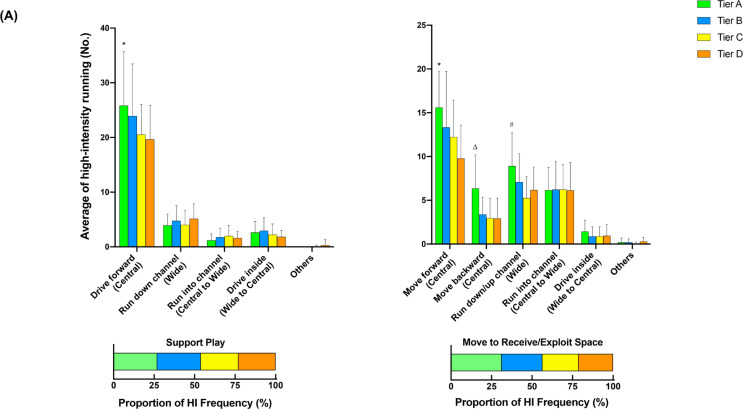
Comparison of additional options for in- (A, B and C) and out-of-possession (D and E) variables between Tiers. Symbols denote differences (P < 0.05). ΔMore actions performed than all other Tiers. *More actions performed than Tier D. #More actions performed than Tier C and D. ●More actions performed than Tier C. ◆More actions performed than Tier B and C.

**FIG. 2b:**
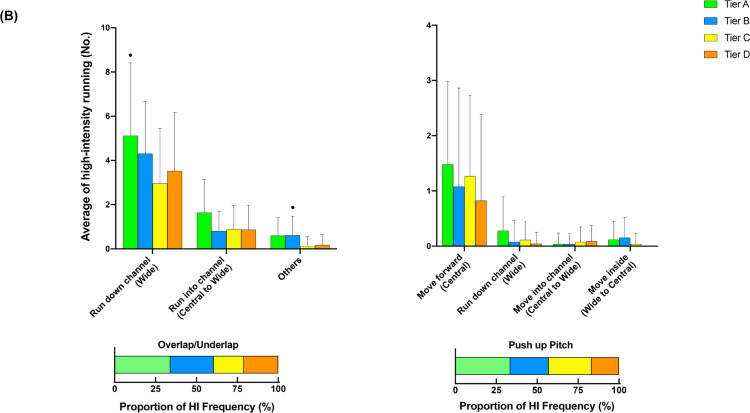
Comparison of additional options for in- (A, B and C) and out-of-possession (D and E) variables between Tiers. Symbols denote differences (P < 0.05). ΔMore actions performed than all other Tiers. *More actions performed than Tier D. #More actions performed than Tier C and D. ●More actions performed than Tier C. ◆More actions performed than Tier B and C.

**FIG. 2c:**
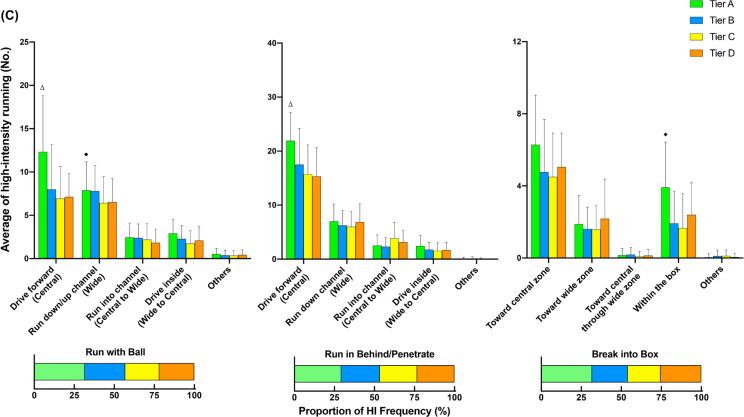
Comparison of additional options for in- (A, B and C) and out-of-possession (D and E) variables between Tiers. Symbols denote differences (P < 0.05). ΔMore actions performed than all other Tiers. *More actions performed than Tier D. #More actions performed than Tier C and D. ●More actions performed than Tier C. ◆More actions performed than Tier B and C.

**FIG. 2d:**
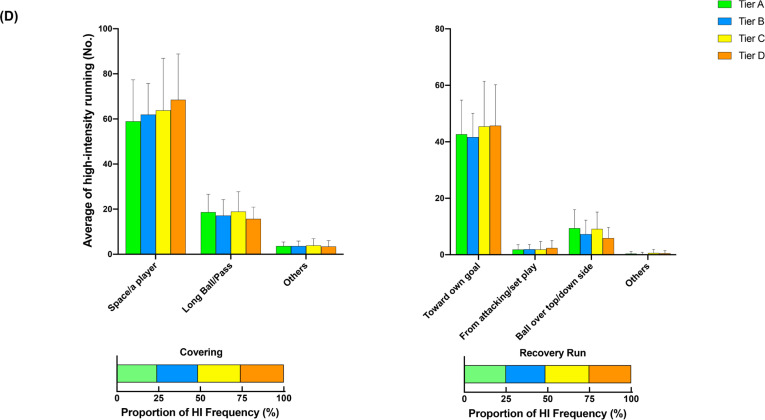
Comparison of additional options for in- (A, B and C) and out-of-possession (D and E) variables between Tiers. Symbols denote differences (P < 0.05). ΔMore actions performed than all other Tiers. *More actions performed than Tier D. #More actions performed than Tier C and D. ●More actions performed than Tier C. ◆More actions performed than Tier B and C.

**FIG. 2e:**
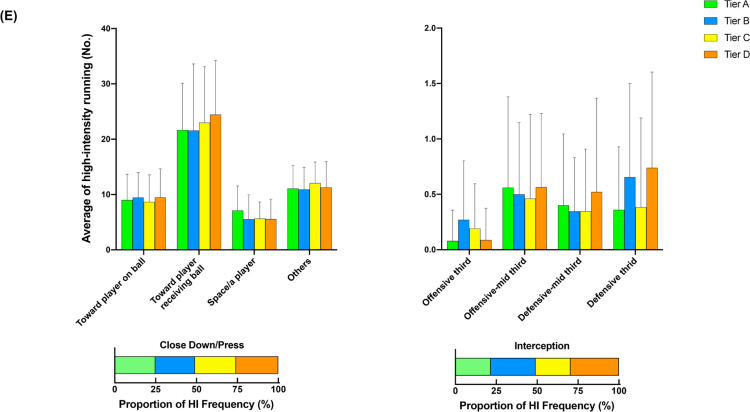
Comparison of additional options for in- (A, B and C) and out-of-possession (D and E) variables between Tiers. Symbols denote differences (P < 0.05). ΔMore actions performed than all other Tiers. *More actions performed than Tier D. #More actions performed than Tier C and D. ●More actions performed than Tier C. ◆More actions performed than Tier B and C.

**FIG. 3a:**
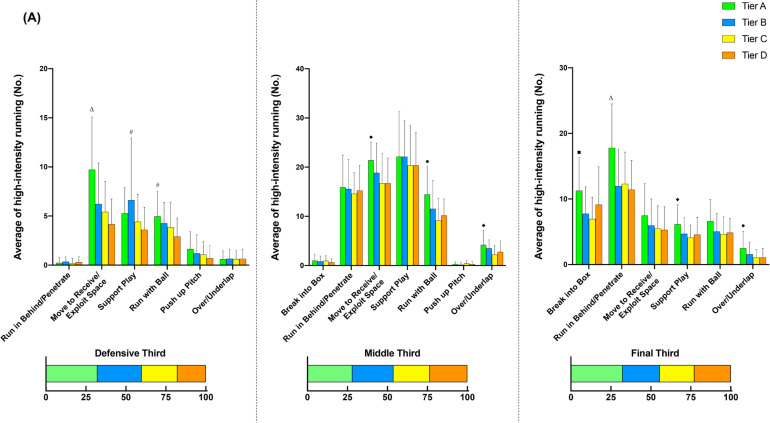
Frequency of high-intensity running in relation to (A) in-possession and (B) out-of-possession categories with special reference to different zones (defensive, middle, and final third). Symbols denote differences (P < 0.05). ΔMore actions performed than Tiers. #More actions performed than Tier D. *More actions performed than Tier A. ●More actions performed than Tier C and D. ◆More actions performed than Tier C. ■More actions performed than Tier B and C.

**FIG. 3b:**
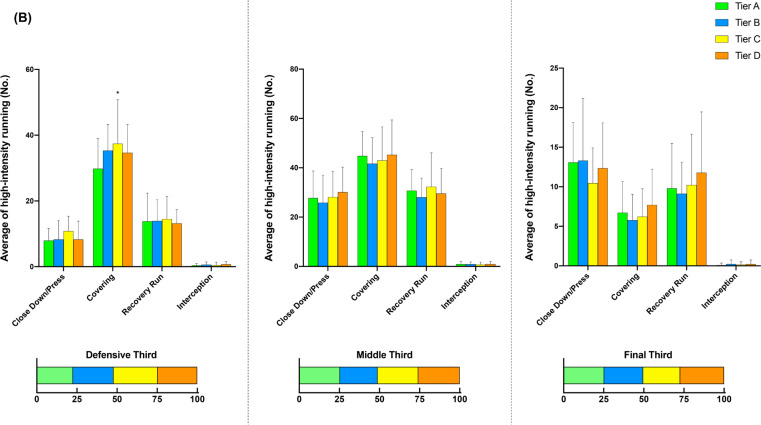
Frequency of high-intensity running in relation to (A) in-possession and (B) out-of-possession categories with special reference to different zones (defensive, middle, and final third). Symbols denote differences (P < 0.05). ΔMore actions performed than Tiers. #More actions performed than Tier D. *More actions performed than Tier A. ●More actions performed than Tier C and D. ◆More actions performed than Tier C. ■More actions performed than Tier B and C.

### Correlation matrix within physical-tactical actions

Regarding ‘within’ dualities (i.e., teammates performing together, Table 3), producing high-intensity actions for ‘Run with Ball’ was highly associated with teammates performing high-intensity ‘Move to Receive/Exploit Space’ (*r* = 0.5, P < 0.01). Regarding ‘between’ dualities (i.e., Team A vs Team B, Table 4), one team performing high-intensity actions of ‘Support Play’ was largely correlated to the opposition team producing high-intensity ‘Recovery Run’ actions (*r* = 0.6–07, P < 0.01).

**TABLE 3:** The Correlation matrix of ‘within’ dualities for physical-tactical actions.

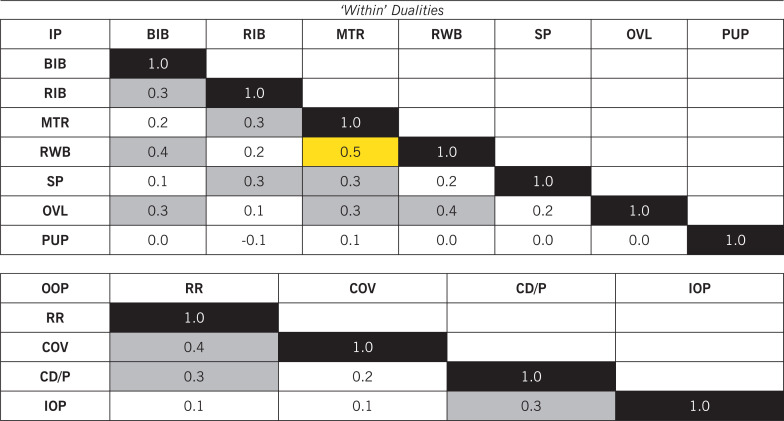

BIB: Break into Box; RIB: Run in Behind/Penetrate; MTR: Move to Receive/Exploit Space; RWB: Run with Ball; SP: Support Play; OVL: Over/Underlap; PUP: Push up Pitch; RR: Recovery Run; COV: Covering; CD/P: Close Down/Press; IOP: Interception. The magnitudes of the correlation coefficients were regarded as trivial (r ≤ 0.1), small (r > 0.1–0.3), moderate (r > 0.3–0.5), large (r > 0.5–0.7), very large (r > 0.7–0.9), and nearly perfect (r > 0.9) [26]. Moderate and large correlations are highlighted in grey (r > 0.3) and orange (r > 0.5–0.7), respectively. IP: In Possession; OOP: Out of Possession.

**TABLE 4:** The correlation matrix of ‘between’ dualities for physical-tactical actions.

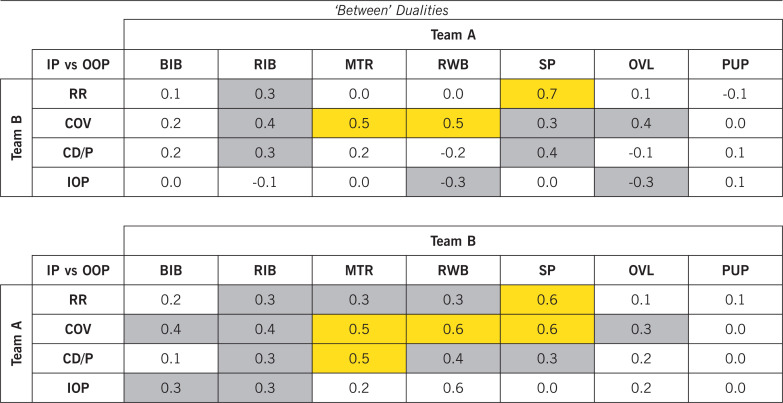

BIB: Break into Box; RIB: Run in Behind/Penetrate; MTR: Move to Receive/Exploit Space; RWB: Run with Ball; SP: Support Play; OVL: Over/Underlap; PUP: Push up Pitch; RR: Recovery Run; COV: Covering; CD/P: Close Down/Press; IOP: Interception. The magnitudes of the correlation coefficients were regarded as trivial (r ≤ 0.1), small (r > 0.1–0.3), moderate (r > 0.3–0.5), large (r > 0.5–0.7), very large (r > 0.7–0.9), and nearly perfect (r > 0.9) [26]. Moderate and (very) large correlations are highlighted in grey (r > 0.3–0.5) and orange (r > 0.5–0.9), respectively. Team A: Home team; Team B: Away Team. IP: In Possession; OOP: Out of Possession.

### Technical performances

**FIG. 4:**
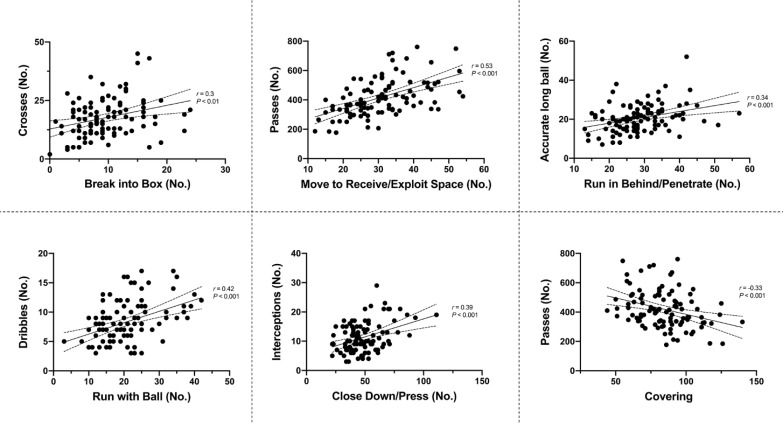
Correlation between physical-tactical (x axis) and technical (y axis) variables. Dotted lines indicate 95% confidence intervals.

Teams in Tier A had 36–57% more shots on target than other Tiers (ES: 0.7–1.2, *P* < 0.05) whilst also completing 24–34% more ball touches and 38–55% more passes (ES: 1.5–1.8, *P* < 0.01). Tier A teams also had a higher passing accuracy (82 ± 5%) than Tier B (78 ± 6%, ES: 0.8, *P* < 0.05) and Tiers C and D (74 ± 5% and 73 ± 7%, respectively, ES: 1.4, *P* < 0.01). The correlations between technical metrics and contextualised actions are presented in Figure 4.

### Match-to-match variabilities of team performance

The mean percentage of CVs in high-intensity distances produced by teams was 13 ± 4%. Regardless of physical-tactical variables, the mean percentage of CVs for the contextualised actions was 48 ± 31%.

## DISCUSSION

The present study is the first to evaluate the physical-tactical trends of elite football teams and players according to their final league ranking. Tier A teams performed more contextualised actions (e.g., ‘Move to Receive/Exploit Space’, ‘Run with Ball’, etc.) as well as better technical skills (e.g., greater number of shots on target, passes, etc.) compared to those in lower Tiers. Regarding positional trends, CDP and WDP in Tier A ran ~65–550% more high-intensity ‘Move to Receive/Exploit Space’ distance than other Tiers. Moreover, the additional options within the physical-tactical actions and zonal differences exhibited more meaningful insights. These data trends aid our understanding of patterns of play according to final league ranking and the discriminatory factors between Tiers.

Data demonstrates that the total high-intensity distances covered by teams in various Tiers were comparable to others (~7100–7800 m vs 7500 m) [28], exhibiting no differences between Tiers. This contrasts previous studies where lower-ranked teams covered greater total distance in high-intensity running compared to higher-ranked teams [8, 9]. This disparity could be due to the different methodological approach applied in the present study (i.e., team performance rather than individual players). Despite this, high-intensity distance covered in possession revealed meaningful differences, which is supported by previous findings [8–10]. Tier A teams covered ~35% more high-intensity distance when they were in possession compared to those in Tier C and D with only ~5% difference for the distance covered when out of possession. Thus, high-intensity distance covered in possession seems to be an important differentiator between team standards in competitions such as the EPL.

Limited evidence exists in the scientific literature to understand ‘WHY’ and ‘HOW’ high-ranked teams cover greater high-intensity distance when in possession. Current findings indicate that although none of the out-of-possession physical-tactical actions displayed any differences between Tiers, meaningful differences were observed regarding in-possession physical-tactical movements. For instance, Teams in Tier A performed ~20–95% more high-intensity distance performing ‘Move to Receive/Exploit Space’, ‘Run with Ball’, ‘Over/Underlap’, ‘Run in Behind/Penetrate’, and ‘Break into Box’ activities compared to lower Tier teams. This clearly explains ‘WHY’ more high-intensity distance is covered by top-ranked teams when in possession than their lower-ranked counterparts. Such contextualised actions could be the actions that higher-standard teams perform more frequently whilst keeping the ball to exploit space whereby producing a viable attacking threat and ultimately scoring a goal [13, 29]. Therefore, these contextualised actions could be key in discriminating between team standards. An important caveat is that this data is limited to the EPL. Therefore, verification of such trends across different competitive standards or other elite football leagues is necessary as different playing styles are expressed in each competition [6, 30].

Literature reports position-specific characteristics with attackers covering ~70–90% more distance at high-intensity in possession compared to out of possession whilst defenders performed ~60–160% greater distance out of possession compared to in possession [8, 30]. However, this provides only rudimentary insights, which may explain why such data are hardly used within the applied setting [19]. In contrast, the present study provides important insights into individual physical-tactical characteristics across Tiers. For instance, whilst in possession CDP and WDP in Tier A covered ~65–550% more high-intensity distances for ‘Move to Receive/Exploit Space’ than those in other Tiers whilst WDP in Tier A ran ~70–80% more distances for ‘Over/Underlap’ and ‘Run in Behind/Penetrate’ than those in Tier C. This agrees with previous findings where CDP and WDP from higher-ranked teams produced more attacking- and passing-related events than their counterparts from lower-ranked teams [31, 32]. This type of data provides clear insights into ‘WHY’ players within high-ranked teams cover more distance at high-intensity when in possession. However, when accounting for relative distance (m/min) covered for each tactical activity as a team using effective playing time (i.e., time of ball in play) which can be affected by team playing style as well as contextual factors [33], no differences were observed between Tiers. This indicates that such trend appears to be simply due to the team having a higher percentage of ball possession during matches. Hence, it would be more beneficial if investigating how effective the physical-tactical actions are during matchplay (e.g., did the action create or nullify a chance/threat?). Additionally, it would be of interest to examine how team’s physical-tactical performances change pertaining to match status and/or opponent standards since they impact match performance [2, 21].

Data analysed from the additional options within the physical-tactical actions demonstrates that in possession Tier A teams noticeably dominated the central area, producing more high-intensity efforts for ‘Support Play’, ‘Move to Receive/Exploit Space’, ‘Run with Ball’, and ‘Run in Behind/Penetrate’ than other Tiers. Interestingly, Tier A clubs also performed more ‘Move to Receive/Exploit Space’ actions while moving backwards compared to other Tiers. For instance, forwards could move back towards their own goal to receive the ball (known as ‘Coming Short’) or defenders could move back and wide to receive the ball when the ball is played to the goalkeeper during build-up play (known as ‘Splitting’). This seems to be due to high-ranked teams more likely adapting a build-up playing style while having a high percentage of ball possession [10, 11, 32]. The analysis of technical data from this study also confirms this notion in which Tier A clubs completed ~25–55% more ball touches and passes than other Tiers, which agrees with previous observations [10, 31]. Although some studies indicate that the EPL teams tend to utilise a counter attack strategy with fast and direct attacks to transition [34, 35], present data demonstrate that EPL teams in the top Tier are more likely to apply a more intricate build-up and possession-based style of play. Nevertheless, as the playing style of teams in top-class teams also differs [36], individual team analysis is warranted to more precisely determine how each team physically and tactically plays during matches. Additionally, as this integrated approach can reveal teams’ playing styles, performance analysts within the team could be benefited from using this approach, especially for opponent analyses, which takes a huge part of the match analysis in football [37].

Distinct differences in physical-tactical actions performed in different zones of the pitch by the diffferent Tiers were apparent. In possession, Tier A teams produced ~30–130% more high-intensity ‘Move to Receive/Exploit Space’ actions from the defensive and middle third compared to lower Tier counterparts. Although previous reports noted that top-ranked teams dominated transition phases [38], they failed to determine what types of tactical actions are critical during this phase of play. Since top-ranked clubs tend to achieve more width and length whilst increasing the offensive play-space than lower-ranked counterparts after regaining the ball [28, 39], this seems to be ‘Move to Receive/Exploit Space’ actions that high-ranked teams perform more often during the defence-to-attack transition phase (e.g., from defensive and middle third) compared to lower-ranked teams. Furthermore, Tier A teams completed ~90–130% more high-intensity ‘Over/Underlap’ actions from the middle and final third than lower Tier teams whilst also performing ~45–60% more actions for ‘Break into Box’ and ‘Run in Behind/Penetrate’ in the final third. This clearly shows ‘HOW’ teams in the top Tier dominated opposition in the middle and final third of the pitch. However, since the present study did not include the phases of play, future research should condense such physical-tactical actions into phases of play to provide extra granularity to match analysis [40].

Out of possession, lower-ranked teams such as those in Tier C demonstrated ~25% more high-intensity activities for ‘Covering’ from the defensive third than higher-ranked teams such as those in Tier A. This action is essential for the team’s defensive organisation whilst being goal side of the ball [18, 41]. Therefore, it seems that low-ranked teams tend to focus on the team’s defensive stability in the defensive third while being goal side, rather than pressing higher up the pitch, which may explain why teams with a defensive formation (e.g., 4-5-1 formations) cover greater distance when out of possession [5]. In contrast, although there were no statistical differences in the frequency of ‘Closing Down/Press’ actions performed by each Tier in the final third, high-ranked teams executed ~20% more of these actions than low-ranked counterparts (13 vs 11). This could indicate that higher-ranked teams are more likely to try to regain the ball higher up the pitch and to counter press if they lose it. Since regaining ball possession in the opposition’s half is important for a team’s success [42], this physical-tactical action seems very promising to evaluate team performance. However, contextual factors such as match status and match location could alter team playing style during match-play [16].

This is the very first time that the relationships of ‘within’ (teammates performing together) and ‘between’ (Team A vs Team B) dualities have been quantified; thus, this provides novel insights into the interactional aspects of physical-tactical components. Producing high-intensity actions for ‘Run in Behind/Penetrate’ was moderately related to teammates performing ‘Support Play’ with the opposition executing ‘Recovery Run’, ‘Covering’, and ‘Close Down/Press’. Additionally, large to very large correlations were found between one team producing ‘Support Play’ and the other team performing ‘Recovery Run’ actions. This could be because when a fast transition occurs, for example, the ball is rapidly moved forward, teammates perform ‘Support Play’ to become involved in the transition or attacking phase with the opponent performing ‘Recovery Run’ actions [13]. Thus, it could be reasonably concluded that one team’s collective behaviour influences the opposition team’s performance and their own team’s tactical behaviour. Nevertheless, further insight may be gained if investigating the individual antagonistic correlations between selected players (e.g., the actions performed by forwards vs those performed by centre backs of the opposition team). Therefore, future research should examine this aspect.

Tier A clubs produced better technical performance such as greater number of shots on target and passes as well as a higher pass accuracy than other Tiers, which agrees with previous findings [9, 14, 15]. This is possibly due to high-standard teams demonstrating higher levels of technical performance whilst also performing greater number of technical events compared to lower-standard teams [9, 10]. Additionally, the present study found that some technical metrics (i.e., OPTA Sports data) were moderately associated with some physical-tactical actions. High-intensity ‘Break into Box’ and ‘Run in Behind/Penetrate’ actions were moderately associated with the number of technical skills such as crosses and accurate long passes, respectively. It may be due to players producing a high-intensity effort trying to enter the opposition box typically expecting a cross from a wide player or to run in behind/penetrate whilst an accurate long ball is being delivered from a deeper player [41]. Moreover, the number of interception events (i.e., technical data) was moderately linked to high-intensity ‘Close Down/Press’ actions. Explanation may reside with the view point that aggressive pressing (e.g., closing down at high-intensity) is able to force the opposition to make mistakes such as inaccurate passes [43]. Collectively, the physical-tactical data appears to be associated with technical metrics available to professional football clubs; thus, this may be more practical for coaches as the context adds a narrative to the data trends. Practitioners are also moving towards an enhanced ability to quantify the impact of the physical work executed by the team on technical and tactical outcomes. Hence, this type of analysis is key to help drive forward physical requirement of the elite player. However, the complex nature of football where numerous contextual factors impact performance during match-play [16], results in high levels of data variability of team performances (e.g., high-intensity distance and contextualised actions: ~13% and ~48%, respectively), thus practitioners should consider these variabilities when making decisions on the practical application of the data.

### Limitations

Firstly, although the present study has integrated physical and tactical performances, this did not integrate ‘technical’ metrics but rather aggregated within the result. As physical, tactical, and technical parameters are fused to influence match performance, future research should amalgamate all these aspects to provide a comprehensive understanding of the true football match performance. Also, another limitation would be a lack of contextual variables included in the study. Therefore, the physical-tactical profiles with special reference to the standard of opposition or match status may be of interest since they have an influence on match performance [2, 21].

## CONCLUSIONS

The contextualised data can help improve the understanding of team playing style and could be used to better discriminate between team standards together with technical metrics. Additionally, players’ physical-tactical actions have an influence on not only their teammates but also opposition’s activities during match-play. However, it should be acknowledged that the match-to-match variabilities in high-intensity distance and contextualised actions are high.
